# Regulatory Functions and Stress-Response Mechanisms of the Plant bHLH Transcription Factor Family

**DOI:** 10.3390/biology15212005

**Published:** 2026-11-05

**Authors:** Zesen Lai, Jifen Yang, Yuhong Rong, Shen Xiong, Yanyin Xu, Zhaohao Zhang, Jiang Xie, Ziyu Li, Huifang Yuan, Xiangshuai Yan, Hongjun Mu

**Affiliations:** Yunnan Institute of Tropical Crops, Jinghong 666100, China

**Keywords:** bHLH transcription factor, abiotic stress, hormone signaling, secondary metabolism, regulatory network, reproductive development

## Abstract

Plant basic helix-loop-helix (bHLH) transcription factors constitute one of the largest and most functionally diverse transcription factor families in plants. Through a conserved bHLH domain, they bind E-box cis-elements or dimerize with partner proteins, coupling developmental and metabolic programs to environmental cues. However, a systematic synthesis of their multilayered stress responses remains lacking. Here, we review the structural features, genome-wide distribution, and evolutionary diversification of plant bHLH transcription factors, with emphasis on their regulatory functions under low-temperature, drought, salinity, and iron-deficiency conditions. We further integrate their roles in abscisic acid, jasmonic acid, and light signaling, as well as in anthocyanin and terpenoid biosynthesis, biotic stress, and immune responses. We also discuss emerging evidence linking bHLH factors to reproductive development, particularly anther and pollen formation under heat stress. Mechanistically, we summarize the multilayered regulatory network from four perspectives: transcriptional cascades, post-translational modifications, cooperative regulation by multiple transcription factors, and growth-defense trade-offs. Finally, we highlight current gaps, including limited functional validation in non-model/specialty economic crop species, underexplored combined-stress mechanisms, and lagging breeding translation, and propose future research directions. This review provides an integrated framework for plant stress biology and outlines evidence-based perspectives for future stress-tolerant crop improvement.

## Simple Summary

Plants are constantly exposed to environmental stresses such as cold, drought, and high salt in the soil, which can severely limit crop growth and yield. To cope, plants rely on proteins that act as molecular switches to turn genes on or off. One of the largest groups of these regulatory proteins in plants is the basic helix-loop-helix (bHLH) family. This review brings together current knowledge about how these proteins recognize specific DNA sequences, work together in pairs, and control the genes that help plants survive cold, drought, salt, and low-iron conditions. It also explains how they connect with plant hormones and light signals, and how they regulate the production of useful compounds such as anthocyanins and terpenoids. We further discuss the emerging roles of bHLH proteins in reproductive development, especially anther and pollen formation under heat stress. By examining how these regulatory proteins coordinate stress responses at multiple levels, we highlight remaining knowledge gaps, such as too little research outside common model plants and a limited understanding of combined stresses, and outline future research directions. A deeper understanding of this protein family can guide the breeding of more stress-tolerant crops, supporting food security under a changing climate.

## 1. Introduction

Plants are sessile organisms that cannot escape adverse environments and must therefore adapt by remodeling their gene expression programs through intricate transcriptional regulatory networks, in which transcription factors act as core nodes linking environmental cues to developmental decisions. Among these regulators, the basic helix-loop-helix (bHLH) transcription factor family, defined by its highly conserved bHLH domain, ranks among the largest and most functionally diverse transcription factor families in plants and exerts broad regulatory control over growth, development, and stress responses. bHLH family members have been implicated in a wide range of processes, from phytochrome signal transduction and cold or drought stress responses to abscisic acid (ABA) signaling and iron homeostasis, with roles spanning upstream signal perception and transduction as well as downstream physiological outputs [1,2]. With the rapid accumulation of genome sequences, genome-wide identification of the bHLH family has been completed in dozens of species, as exemplified by genome-wide surveys in soybean and maize [3,4]. Phylogenetically, the classical *Arabidopsis*-based system originally recognized 12 subfamilies [5], a framework since refined into 17 major groups comprising 31 subfamilies as genome coverage and phylogenetic resolution have improved [6]; broader multi-species analyses incorporating algae, moss, and multiple angiosperm lineages have resolved the plant bHLH family into up to 32 subfamilies [7]. Together, these frameworks provide a foundation for tracing the evolutionary trajectories of the family and predicting the functions of individual members.

Recent advances in molecular biology and genetics have progressively shifted bHLH research from the characterization of individual genes toward the dissection of regulatory networks. These studies have uncovered roles for bHLH transcription factors in jasmonic acid (JA) signaling, secondary metabolite biosynthesis, and the growth–defense trade-off, establishing them as central integrators of multiple signaling inputs [8,9]. Nevertheless, although a wealth of data has accumulated, the current understanding of plant bHLH transcription factors remains fragmentary and uneven across plant lineages and stress types, and a systematic integration of their multilayered regulatory networks, as well as of the functional conservation and diversification of this family across species, is still lacking. This review therefore focuses on the regulatory functions and stress response mechanisms of plant bHLH transcription factors, and integrates the topic from four aspects: family structure and evolution, abiotic stress and hormonal regulation, secondary metabolism and biotic stress responses, and multilayered regulatory networks. We further discuss future directions, aiming to provide a clear theoretical framework and research perspectives for this field.

We performed a systematic literature search to ensure comprehensive and reproducible coverage. PubMed, Web of Science, and China National Knowledge Infrastructure databases were searched from inception up to October 2026 using combinations of the terms “bHLH”, “basic helix-loop-helix”, “transcription factor”, and the stress- or process-related terms covered in this review (cold, drought, salinity, iron, heat, jasmonic acid, light, anthocyanin, terpenoid, pathogen, immunity, anther, pollen). Studies were selected according to the following inclusion criteria: (i) articles focused on plant bHLH transcription factors and their regulatory roles in the stress, developmental, and metabolic processes covered by this review; (ii) original experimental, genome-wide, or comprehensive review evidence; and (iii) peer-reviewed original articles and reviews. Conference abstracts, preprints, and studies without accessible full text were excluded. Representative studies were then selected and organized by stress category, plant lineage, and regulatory level for the thematic sections below.

**Figure 1:**
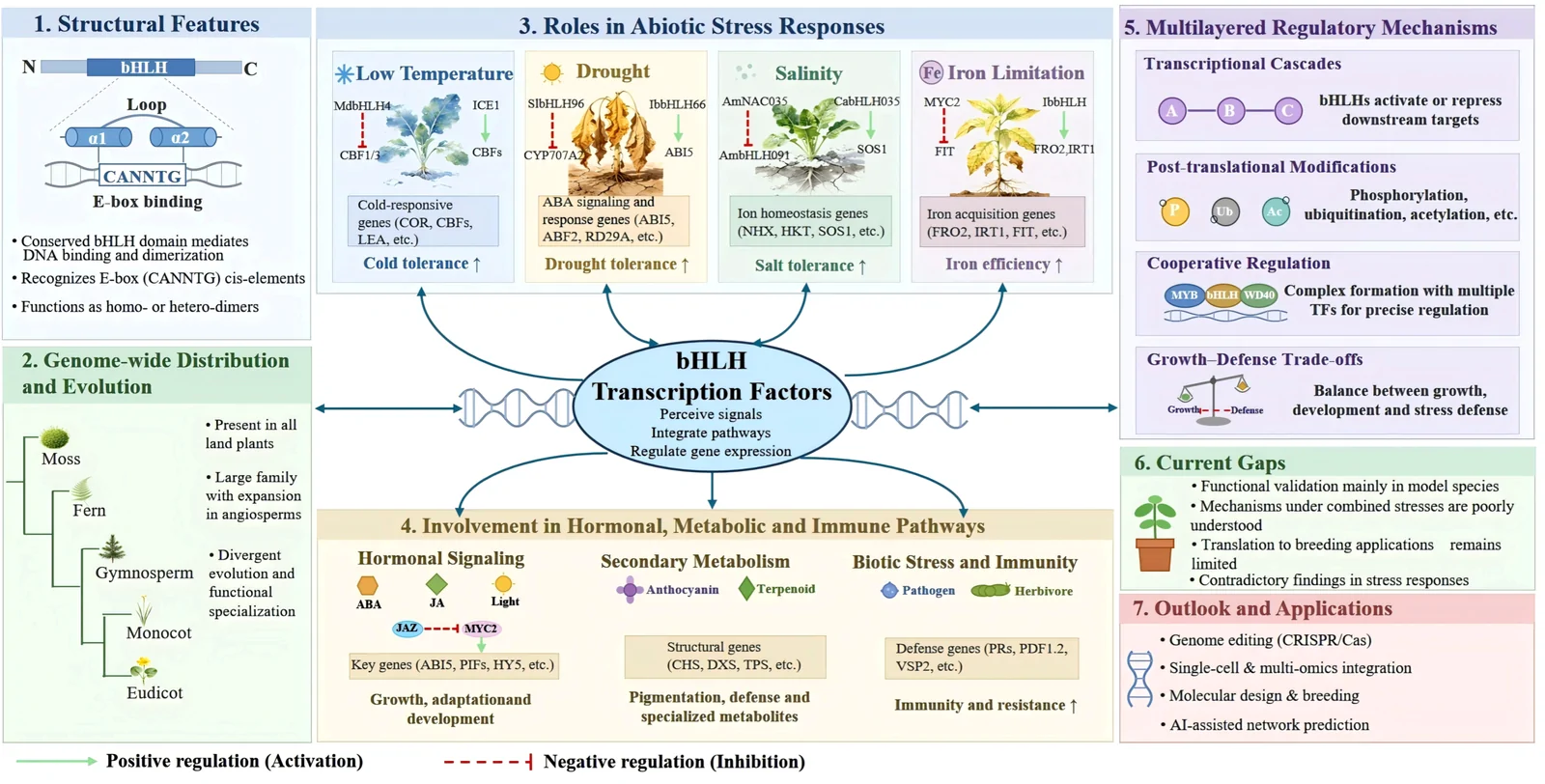
Schematic overview of the regulatory functions and stress-response mechanisms of the plant bHLH transcription factor family. Schematic overview of the principal regulatory connections of the plant bHLH transcription factor family. Environmental and hormonal inputs (cold, drought, salinity, iron limitation, heat, ABA, JA, and light) converge on bHLH transcription factors, which act through multilayered regulatory mechanisms—transcriptional cascades, post-translational modifications, cooperative complex formation, and growth–defense trade-offs—to regulate downstream target genes and phenotypic outputs, including stress tolerance, secondary metabolite accumulation, immunity, and male fertility. In the jasmonic acid signaling module, the JAZ-mediated inhibition of MYC-type bHLH transcription factors is depicted as an inhibitory (red dashed) connection [8,12], whereas the MYB-bHLH-WD40 complex, which cooperatively regulates anthocyanin biosynthesis, is represented under cooperative complex formation rather than as a transcriptional cascade [10,11,13]. Green arrows indicate positive (activating) regulation, and red dashed lines indicate negative (inhibitory) regulation.The upward arrows (↑) indicate an increase or improvement in the corresponding traits.

To provide a systematic framework for the following sections, the major structural, functional, and regulatory themes of plant bHLH transcription factors are summarized in Figure 1. The figure depicts how environmental and hormonal inputs converge on bHLH transcription factors and how multilayered regulatory mechanisms translate these signals into downstream target-gene regulation and phenotypic outputs, all of which are discussed in detail below. In particular, the JAZ-mediated inhibition of MYC-type bHLH transcription factors in jasmonic acid signaling and the cooperative MYB-bHLH-WD40 complex in anthocyanin regulation are explicitly depicted as distinct regulatory modes, with the supporting references [8,10,11,12,13].

Several comprehensive reviews of plant bHLH transcription factors have been published in recent years, each focusing on a particular stress category, taxon, or regulatory level [14,15,16,17,18,19,20]. The present review differs from these previous syntheses in three respects. First, it integrates the complete chain from family structure and evolution to multilayered regulatory networks within a single framework, rather than restricting coverage to abiotic stress alone. Second, it incorporates advances from the past five years, including the hierarchical iron-regulatory cascade and its grass-specific divergence [21,22,23,24], post-translational regulation of tapetal bHLH activity by the SBI1–PP2A module [25], heat-sensitive male-fertility functions of bHLH010/089/091 [26], and the resolution of the growth–defense trade-off in *Artemisia annua* [27]. Third, it explicitly links molecular regulation to future breeding-oriented applications. We believe this integrative perspective constitutes the unique contribution of the present work.

## 2. Structure and Evolutionary Distribution of Plant bHLH Transcription Factors

### 2.1. Protein Structure and Functional Basis

The defining feature of bHLH transcription factors is a conserved bHLH domain of approximately 60 amino acids, comprising two functional regions: a basic region at the N-terminus and a helix-loop-helix (HLH) region at the C-terminus. The basic region, a short N-terminal stretch of about 13–17 amino acids enriched in basic residues, is responsible for recognizing and binding the E-box (5′-CANNTG-3′) cis-element in target gene promoters and constitutes the structural basis for the transcriptional regulatory function of bHLH proteins [28]. The HLH region consists of two amphipathic alpha-helices connected by a loop and mainly mediates protein dimerization, allowing the formation of homo- or heterodimers; different dimer combinations confer distinct DNA-binding specificities and transcriptional activities, representing an important molecular basis for the functional diversification of the bHLH family [10].

Based on the sequence features and DNA-binding capacity of the basic region, plant bHLH proteins can be classified into canonical DNA-binding and noncanonical non-DNA-binding types. Most bHLH proteins belong to the canonical binding type and directly bind target gene promoters to regulate transcription. In contrast, noncanonical bHLH proteins carry key amino acid substitutions in the basic region that abolish DNA binding, and they mainly regulate the expression of downstream genes indirectly by forming heterodimers with other bHLH proteins. When heterodimerizing with DNA-binding partners, some noncanonical bHLH proteins can inhibit the transcriptional activity of the latter, thereby functioning as negative regulators of specific downstream transcriptional programs. The *Arabidopsis* proteins PRE3/bHLH135/ATBS1/TMO7 are typical non-DNA-binding bHLH proteins that participate in the negative regulation of light signaling through protein–protein interactions [29].

In addition to the core bHLH domain, bHLH proteins in specific subfamilies harbor additional functional domains that participate in metabolic signal responses and protein–protein interactions, further expanding the functional repertoire of the bHLH family [28]. Structural studies of the MYB-bHLH transcription factor complex have revealed the molecular basis underlying their interaction selectivity, in which variations in key amino acids at the protein interface determine the interaction specificity of different subfamilies; this structural feature has driven the co-evolution and functional divergence of these two transcription factor families [11].

### 2.2. Genome-Wide Identification and Species Distribution

As plant genome sequences have accumulated, bHLH family surveys now span the full range of plant lineages, from algae and bryophytes to ferns, gymnosperms, and angiosperms. The number of bHLH genes varies markedly among species, with an overall trend toward expansion of family size as plant evolutionary complexity increases [10].

Among model plants, the *Arabidopsis* bHLH family represents the most intensively studied and most comprehensively annotated system to date, and its classification and functional framework has become a reference for studies in other species [6]. Rice bHLH genes have been examined from several angles, including abiotic stress responses and nutrient homeostasis, and rice now serves as a model system for bHLH research in grasses [1,2].

The hexaploid wheat genome carries approximately 571 bHLH genes [30], which diverge markedly in tissue-specific expression and stress responsiveness; some members respond to multiple abiotic stresses, including drought, salinity, and low temperature [31,32]. In maize, bHLH genes can be divided into multiple subfamilies, several of which participate in abiotic stress responses and the regulation of growth and development [4]. The soybean genome harbors a large number of stress-responsive bHLH members whose expression patterns are induced by drought and salinity, providing candidate resources for the mining of stress resistance genes in legumes [3,33,34]. The same survey has been extended to *oat*, *rye*, and *Brachypodium distachyon*, providing genomic resources that complement those available for wheat and other cereals [35,36,37].

In apple (*Malus domestica*), the bHLH family has been systematically identified and characterized, and expression profiling under drought and salinity treatments has yielded numerous candidate genes for stress resistance breeding in fruit trees [38]. Comparable catalogs now exist for tomato (*Solanum lycopersicum*), mango (*Mangifera indica*), sweet cherry (*Prunus avium*), and longan (*Dimocarpus longan*), from which genes involved in salinity tolerance, cold tolerance, and flowering regulation have been screened [39,40,41].

Among the medicinal plants, identification and functional analysis of the bHLH family in *ginseng*, *Scutellaria baicalensis*, and *Angelica sinensis* have mainly focused on the regulation of secondary metabolite biosynthesis, providing a molecular basis for the quality improvement of medicinal plants [42,43]. Stress-adapted species have also drawn attention: genome-wide surveys in sand rice (*Agriophyllum squarrosum*) and crested wheatgrass (*Agropyron cristatum*) have yielded genetic resources for studying stress resistance mechanisms in desert and stress-tolerant plants [44,45] (Table 1).

### 2.3. Family Evolution and Functional Divergence

Phylogenetic and functional analyses based on the classical *Arabidopsis*-derived classification have shown that the plant bHLH family can be divided into 12 subfamilies [5]. Subsequently, with the expansion of species coverage and phylogenetic resolution, current frameworks have further divided the family into 17 major groups comprising 31 subfamilies [6], while broader multi-species analyses have resolved the family into up to 32 subfamilies [7]. During evolution, different subfamilies have undergone pronounced functional divergence: some subfamilies are highly conserved across eukaryotes and mainly regulate the cell cycle and basic developmental processes, whereas others are plant-specific subfamilies that have evolved plant-specific functions, such as the regulation of photosynthesis, stress responses, and secondary metabolism [10].

Functional studies of bHLH transcription factors in liverwort (*Marchantia polymorpha*) have revealed that this family is involved in the origin and evolution of plant stomata and serves as a key regulator of terrestrial adaptation during the water-to-land transition of plants, providing evolutionary evidence for understanding the functional diversification of the bHLH family [68]. Comparative evolutionary analyses of the MYB and bHLH transcription factors have further shown that the two families underwent coordinated expansion during the evolution of land plants, jointly constructing the transcriptional regulatory network underlying plant secondary metabolism and stress responses [10]. Furthermore, the transcriptional complex formed by NGATHA and bHLH transcription factors in *Arabidopsis* specifically regulates stigma development and determines pistil morphogenesis, indicating that the bHLH family has also evolved diversified regulatory functions in the development of reproductive organs [69].

Overall, comparative analyses across plant lineages suggest that the plant bHLH family has diversified through a “conserved domain and variable regulatory region” strategy, although direct evidence linking regulatory-region variation to habitat-specific functional adaptation remains limited and warrants further investigation. This architecture is proposed to preserve the core DNA-binding and dimerization functions while generating diverse stress-responsive functions adapted to distinct habitats through variation in regulatory regions and the expansion of family members; however, direct experimental evidence linking regulatory-region variation to habitat-specific functional adaptation is still required. If validated, this would position the bHLH family as an important genetic resource for plant adaptation to the complex terrestrial environment.

## 3. Regulatory Functions and Hormone Signaling-Mediated Mechanisms of bHLH Transcription Factors in Abiotic Stress

Abiotic stress is a major environmental factor limiting crop yield and geographic distribution. As central regulatory nodes of stress response networks, bHLH transcription factors integrate multiple endogenous signaling pathways, including abscisic acid (ABA), jasmonic acid (JA), and light signaling, and mediate plant adaptive responses to various abiotic stresses, such as low temperature, drought, salinity, iron homeostasis imbalance, and high temperature, at the physiological, biochemical, and morphological levels by regulating the expression of downstream functional genes.

Hormonal and light signaling integrate multiple abiotic stress responses, and the corresponding bHLH regulatory modules are introduced here because they function as cross-stress frameworks. Within the jasmonic acid signaling pathway, the MYC subfamily of bHLH transcription factors serves as the core downstream regulator: JAZ (JASMONATE ZIM-DOMAIN) proteins can directly interact with bHLH proteins and inhibit their transcriptional activity, and upon stress, jasmonate accumulation induces the degradation of JAZ proteins, releasing bHLH activity to initiate the expression of downstream stress-responsive and secondary metabolism genes. The JAZ-bHLH regulatory module thus constitutes the central framework of the jasmonic acid signaling pathway in plants, mediating the complete process from hormonal signal transduction to downstream physiological responses [8,12]. In the light signaling pathway, the PIF (PHYTOCHROME-INTERACTING FACTOR) subfamily of bHLH transcription factors can directly interact with phytochrome proteins, mediating the regulation of plant gene expression by light signals, and can integrate light and endogenous hormonal signals to coordinately regulate plant development and stress adaptation [70,71]. Studies of CsPIF7 and CsbHLH89 in tea plants (*Camellia sinensis*) have further revealed the functions of the PIF family in light signal transduction and secondary metabolism regulation in woody plants [72,73] (Table 2).

Low-temperature injury is a major constraint on tropical and subtropical crop production, and bHLH transcription factors lie at the core of the signal transduction and acclimation responses that plants mount against it. In *Arabidopsis*, ICE1 (INDUCER OF CBF EXPRESSION 1) is an important cold-responsive bHLH transcription factor that binds to and activates the promoters of CBF (C-REPEAT BINDING FACTOR) cold-responsive genes, constituting the ICE1-CBF core regulatory module that mediates plant cold acclimation [74]. ICE1 homologs (e.g., OsICE1) and conserved ICE-CBF regulatory modules have also been identified in grasses such as rice and wheat, indicating that this pathway is highly conserved across angiosperms. *OsbHLH1* from rice is one of the earliest identified cold-responsive bHLH genes in this species, and its expression is specifically induced by low temperature, suggesting that it may participate in cold signal transduction as a transcription factor [1]. Subsequent studies in woody and herbaceous plants, including paper mulberry (*Broussonetia papyrifera*), sweet cherry (*Prunus avium*), alfalfa (*Medicago sativa*), Siberian apricot (*Prunus sibirica*), and *Liriodendron chinense*, have shown that multiple bHLH family members undergo significant expression changes under low-temperature treatment, identifying candidate members whose stress-responsive expression suggests possible involvement in cold acclimation, pending genetic and biochemical validation [52,59,62,63,75]. Mechanistically, bHLH transcription factors regulate cold tolerance through three main pathways: first, directly binding and activating cold-responsive genes to enhance cellular osmotic adjustment; second, regulating genes of the antioxidant system to alleviate cold-induced oxidative damage; and third, indirectly affecting cold responses by modulating hormone signaling pathways, such as ABA and JA. The regulatory pathways in different species exhibit both conservation and species-specific divergence [14]. Very recently, IbbHLH129 from sweet potato (*Ipomoea batatas*) was shown to positively regulate seedling cold tolerance by modulating the auxin- and gibberellin-related pathways, extending functional validation of cold-responsive bHLH factors to tuber crops [76].

Drought ranks among the most damaging stresses for agriculture worldwide. bHLH transcription factors mitigate its effects through several physiological routes, including stomatal movement, root development, ABA homeostasis, and reactive oxygen species (ROS) scavenging, with the ABA signaling pathway serving as the central regulatory route for these responses. bHLH transcription factors play a dual role in the ABA pathway: they act as downstream effectors of ABA signaling to execute stress responses, and they can also negatively modulate hormone signal intensity by regulating ABA metabolic homeostasis, thereby achieving the precise control of stress responses [46,77]. *AtbHLH68* in *Arabidopsis* regulates stomatal movement and growth status under drought by directly or indirectly modulating the expression of key genes involved in ABA biosynthesis and metabolism to maintain ABA homeostasis, and loss of its function significantly increases plant drought sensitivity [78]. ZmPTF1 in maize promotes ABA biosynthesis and root development under drought conditions, significantly increasing the plant survival rate under drought [49]. With respect to stomatal and oxidative homeostasis, SlbHLH96 in tomato (*Solanum lycopersicum*) and MdbHLH130 in apple (*Malus domestica*) enhance plant resistance to water stress by regulating stomatal closure and ROS homeostasis, directly activating the expression of antioxidant enzyme genes to alleviate oxidative damage while promoting stomatal closure to reduce water loss [53,57]. In terms of root development, MdCIB1 in apple (*Malus domestica*), PxbHLH02 in poplar (*Populus*), and CgbHLH001 in *Chenopodium glaucum* confer stronger drought tolerance by positively regulating root morphogenesis and the expression of stress-responsive genes [58,60,66]. Furthermore, the identification of species-specific genes, including OsWIH2 in rice, AhbHLH112 in peanut (*Arachis hypogaea*), and RERJ1 in rice, has further enriched our understanding of the drought-regulatory functions of bHLH transcription factors across different plant lineages [79,80,81]. Genome-wide analysis of the bHLH family in *Populus trichocarpa* has confirmed that multiple members participate in drought stress responses, providing genetic resources for the molecular breeding of drought resistance in woody plants [61].

The dual constraints of osmotic stress and ion toxicity make salt one of the harshest abiotic challenges for plant growth. bHLH transcription factors improve salt tolerance by regulating root morphogenesis, ion homeostasis, and osmotic adjustment, and these functions are tightly coupled to hormone signaling pathways. *SbbHLH85* in sorghum (*Sorghum bicolor*) is a key bHLH gene regulating root hair development; its overexpression increases root hair number and length, which can enhance Na^+^ accumulation under salt stress and thereby reduce salt tolerance, making SbbHLH85 a context-dependent negative regulator of salt tolerance in sorghum [50]. AhbHLH121 in peanut improves salt stress tolerance by regulating Na^+^/K^+^ ion homeostasis and the antioxidant system, maintaining a lower Na^+^/K^+^ ratio to alleviate ion toxicity [82]. PdbUNE12 in poplar acts as a positive regulator of salt stress responses and enhances plant salt tolerance by activating the expression of salt stress-responsive genes, representing an important genetic resource for the salt-tolerant genetic improvement of forest trees [83]. Genome-wide expression profiling has shown that multiple bHLH members respond to salt stress treatment in species such as *Brachypodium distachyon*, *Cyclocarya paliurus*, tomato (*Solanum lycopersicum*), *Hibiscus hamabo*, and castor bean (*Ricinus communis*), with different members exhibiting differences in response timing and expression magnitude. Given the absence of experimental evidence for direct regulatory interactions, these genes are currently best described as salt stress-responsive candidates [36,39,84,85,86].

Although iron is essential for plant growth, it is rarely present in soil in a form that plants can use. Plants therefore maintain iron homeostasis through a regulatory network in which bHLH transcription factors are core components, arranged in a characteristic hierarchical cascade. The first iron-regulatory bHLH identified in plants was the FER gene in tomato, which opened the era of functional studies on bHLH-mediated iron homeostasis [87]. In grasses, *IRO2* is a core iron-regulatory bHLH gene whose expression is strongly induced by iron deficiency; it regulates the expression of genes involved in mugineic acid biosynthesis, promoting the chelation and uptake of ferric iron [2]. OsFIT/OsbHLH156 (FER-LIKE IRON DEFICIENCY-INDUCED TRANSCRIPTION FACTOR) further interacts with IRO2 to promote its nuclear translocation and transcriptional activation, as demonstrated by protein–protein interaction and subcellular localization assays. Studies in *Arabidopsis* have further revealed the hierarchical cascade network: bHLH34, bHLH104, bHLH105, and bHLH115, which belong to clade IVc, act as upstream regulators that respond to iron deficiency signals and directly activate the expression of downstream clade Ib bHLH genes (bHLH38/39/100/101), as confirmed by chromatin immunoprecipitation (ChIP) and yeast one-hybrid assays; these clade Ib proteins in turn interact with the central regulator FIT to regulate the transcription of functional genes involved in iron uptake and transport via yeast two-hybrid and bimolecular fluorescence complementation (BiFC) analyses; together, genetic and DNA-binding experiments have established the directness of these hierarchical interactions, jointly maintaining iron homeostasis in plants [21,22]. In contrast to the *Arabidopsis* model, rice has evolved lineage-specific negative regulators such as OsIRO3, which directly repress the expression of the iron-regulatory bHLH transcription factor OsIRO2 to prevent iron overload, and OsbHLH062, a newly characterized negative regulator that recruits TOPLESS corepressors to repress OsIRO2 transcription as validated by protein interaction assays [88]. In crops, OsbHLH057 in rice and SlbHLH152 in tomato participate in regulating iron uptake, transport, and homeostasis maintenance [47,54]. OsbHLH064 in rice not only regulates iron homeostasis but also promotes iron enrichment in grains, providing a key target for iron biofortification breeding in crops [48]. Overall, the iron-regulatory bHLH pathways in dicots and grasses share a conserved hierarchical architecture [21,22,23] but differ in key regulatory components: rice possesses dedicated negative regulators and FIT-dependent nuclear translocation, whereas dicot species show species-specific divergence in signal-responsive components and downstream target gene repertoires. Whether the rice-specific regulatory features are shared by other grass species remains to be experimentally confirmed.

In addition to the core abiotic stresses described above, bHLH transcription factors also participate in responses to other stresses, including high temperature and heavy metals. The jasmonic acid and light signaling modules that mediate cross-integration of different stress responses are introduced at the beginning of this section (Table 2). Under high-temperature stress, CsSPT in cucumber (*Cucumis sativus*) improves plant thermotolerance by regulating the expression of genes related to photosynthesis and chloroplasts [55]. In *Rhododendron hainanense*, bHLH members such as RhbHLH107 and RhbHLH49 have been verified as positive regulators of thermotolerance, and thermotolerance-related candidate genes, including RsbHLH53 and RsbHLH59, have also been screened in rhododendron [89,90]. In the response to cadmium-induced oxidative stress, bHLH family members alleviate the oxidative damage and toxicity caused by cadmium stress by regulating the expression of genes involved in the antioxidant system and detoxification [91].

To provide a structured overview of these studies, Table 2 summarizes representative bHLH transcription factors involved in abiotic stress responses, providing information on the species, expression pattern, and regulatory mechanism of each locus, and specifying whether each entry represents a functionally validated regulator or an expression-based candidate.

**Table 2:** Key bHLH transcription factors involved in abiotic stress responses.

Stress Type	Gene/Locus	Species	Encoded Protein	Expression Pattern	Regulatory Mechanism	References	Evidence Level
Cold stress	*OsbHLH1*	*Oryza sativa*	bHLH TF	Whole-plant expression under cold, upregulated in seedlings	Cold-induced expression, participates in cold signal transduction	[1]	Expression-based candidate
	*PavbHLH16/42*, etc.	*Prunus avium*	bHLH TF	Rapid response in leaves and flower buds under early cold	Differential expression under cold treatment	[59]	Expression-based candidate
Drought stress	*AtbHLH68*	*Arabidopsis thaliana*	bHLH TF	Upregulated in roots and leaves under drought	Regulates ABA biosynthesis and catabolism, maintains ABA homeostasis	[78]	Functionally validated
	*ZmPTF1*	*Zea mays*	bHLH TF	Significantly upregulated in roots under drought	Promotes root development and ABA synthesis, enhances survival rate	[49]	Functionally validated
	*MdbHLH130*	*Malus domestica*	bHLH TF	Upregulated in leaves and guard cells under water stress	Regulates stomatal closure and ROS homeostasis, alleviates oxidative damage	[57]	Functionally validated
Salt stress	*SbbHLH85*	*Sorghum bicolor*	bHLH TF	Specifically upregulated in root hairs under salt	Promotes root hair elongation but negatively regulates salt tolerance via Na^+^ overaccumulation	[50]	Functionally validated
	*AhbHLH121*	*Arachis hypogaea*	bHLH TF	Upregulated in roots and leaves under salt	Regulates Na^+^/K^+^ homeostasis and antioxidant system, alleviates ion toxicity	[82]	Functionally validated
Iron homeostasis	*IRO2*	*Oryza sativa*	bHLH TF	Strongly induced in roots under iron deficiency	Regulates mugineic acid synthesis genes, promotes Fe(III) chelation and uptake	[2]	Functionally validated
	*bHLH34/bHLH104*	*Arabidopsis thaliana*	bHLH TF	Upregulated in roots under iron deficiency	Upstream hierarchical regulators activate downstream bHLH gene expression	[21,22]	Functionally validated
	*OsbHLH064*	*Oryza sativa*	Clade IVb bHLH	Expressed in roots and grains	Regulates iron uptake/transport, promotes grain iron accumulation	[48]	Functionally validated

When compared across stress systems, bHLH-mediated regulation shows both shared and stress-specific features. Shared mechanisms include the coupling of bHLH activity to ABA and JA signaling, the regulation of ROS-scavenging and osmotic-adjustment genes, and the use of hierarchical transcriptional cascades and post-translational modifications [14,92]. Distinct features include the ICE1–CBF module for cold acclimation, ABA-centered stomatal and root responses for drought, ion-homeostasis networks for salinity, the clade IVc–Ib–FIT cascade for iron acquisition, and the growth–immunity trade-off for pathogen defense [9,21,22,50,74,82]. At the regulatory level, nutrient stresses such as iron limitation rely more on lineage-specific negative regulators, whereas temperature and pathogen stresses more frequently engage inducible positive regulators; this contrast indicates that combined stresses cannot be treated as simple additive responses and highlights the need for multi-stress studies.

## 4. Regulation of Secondary Metabolism and Biotic Stress by bHLH Transcription Factors

### 4.1. Regulation of Secondary Metabolite Biosynthesis

Plant secondary metabolites not only participate in stress defense but also constitute key determinants of crop quality and medicinal value. bHLH transcription factors are important regulators of secondary metabolite biosynthesis, and members of different subfamilies can specifically regulate the synthesis of different types of secondary metabolites, influencing their accumulation by regulating the expression of genes encoding key enzymes in the biosynthetic pathways.

Anthocyanins are among the most intensively studied secondary metabolites, and their biosynthesis is precisely regulated by the MYB-bHLH-WD40 (MBW) protein complex, in which bHLH is an essential component that determines the transcriptional activation activity of the complex. In contrast, the cell elongation regulators LP1 and LP2 have been shown to regulate longitudinal cell elongation [93]; direct experimental evidence for their involvement in secondary metabolite distribution is lacking, and they are therefore not included among the experimentally validated regulators of anthocyanin biosynthesis discussed below. In *gentian* (*Gentiana*), bHLH transcription factors cooperate with R2R3-MYB to regulate anthocyanin biosynthesis and determine the pigment accumulation patterns of floral organs [94]. DcTT8 in *Dendrobium candidum*, NcTT8 in water lily (*Nymphaea colorata*), and FabHLH110 in strawberry (*Fragaria*) have all been confirmed to directly bind the promoters of genes encoding key enzymes of anthocyanin biosynthesis, regulating anthocyanin accumulation, and represent key genes for color quality regulation in medicinal and ornamental plants [13,95,96]. More recently, VvbHLH137 in grape (*Vitis vinifera*) was shown to positively regulate anthocyanin accumulation by interacting with VvMYB15, VvMYB44, and VvMYB306 and directly activating the *VvDFR* and *VvF3H* promoters, extending the validated MBW framework to fruit crops [97].

Regarding the regulation of terpenoid secondary metabolism, the two bHLH transcription factors TSAR1 and TSAR2 in *Medicago truncatula* specifically regulate triterpenoid saponin biosynthesis, promoting saponin accumulation by activating the expression of key genes in the biosynthetic pathway [51]. The transcription factor AabHLH112 in *Artemisia annua* positively regulates the biosynthesis of sesquiterpenes, such as epi-cedrol and beta-farnesene [64]. PatMYC1 in patchouli (*Pogostemon cablin*) regulates sesquiterpene biosynthesis by activating the mevalonate (MVA) pathway, representing an important target for the regulation of active ingredients in medicinal plants [65]. Furthermore, the identification of bHLH members associated with ferulic acid biosynthesis in *Angelica sinensis* and of bHLH genes involved in the regulation of baicalin biosynthesis in *Scutellaria baicalensis* has further expanded the application of bHLH transcription factors in the regulation of secondary metabolism in medicinal plants [43,98]. Transcription factors mediate the accumulation of plant secondary metabolites under stress by regulating the expression of secondary metabolism-related genes, and bHLH proteins constitute an important regulatory class within this process [99].

### 4.2. Regulation of Biotic Stress and Immune Responses

bHLH transcription factors not only participate in abiotic stress responses but also play important regulatory roles in plant biotic stress and innate immunity. The *Arabidopsis* transcription factor HBI1 negatively regulates the pathogen-associated molecular pattern (PAMP)-triggered immune response while promoting plant growth, functioning as a core regulator mediating the growth–immunity physiological trade-off [9].

With respect to disease responses, CsbHLH085 in sweet orange (*Citrus sinensis*) positively regulates resistance to citrus canker by activating the expression of defense-related genes and phytoalexin biosynthetic genes, enhancing plant disease resistance, and represents a potential target for disease resistance breeding in citrus [56]. Transcription factors play core regulatory roles in plant defense signaling pathways, and bHLH family members participate in immune responses by regulating the expression of defense-related genes [100]. During stress responses, the transcriptional regulation of bHLH genes exhibits pronounced spatiotemporal specificity, with significantly different expression patterns across tissues and stress stages, constituting a dynamic transcriptional response network [101]. Overall, the functions of bHLH transcription factors in biotic stress are mainly manifested at two levels: first, acting as regulatory hubs of the growth–immunity balance to coordinate resource allocation; and second, directly regulating defense genes and secondary metabolite biosynthesis to enhance plant resistance to the biotic stresses addressed in this review, in particular PAMP-triggered immunity and citrus bacterial canker resistance (Table 3).

## 5. bHLH Transcription Factors in Reproductive Development and Heat-Sensitive Male Fertility

Beyond their stress-related roles, bHLH factors are also central to plant reproductive development, particularly anther and pollen formation. This section focuses on reproductive development as an emerging intersection between bHLH-mediated regulation and heat stress. The pollen wall—a multilayered extracellular structure that protects microspores and mediates pollen–stigma recognition—is assembled from sporopollenin and lipidic precursors [102]. This biosynthetic program is governed by the tapetum, the innermost anther layer that nourishes developing microspores and supplies structural materials for pollen maturation [103]. It should be noted that the molecular cascade described below has been established predominantly in *A. thaliana*, and the extent to which these mechanisms are conserved in other species, particularly with respect to heat-sensitive male fertility, remains to be determined.

Genetic studies in *Arabidopsis* have uncovered a hierarchical transcriptional cascade governing tapetal function, with bHLH factors occupying central nodes. DYT1 (DYSFUNCTIONAL TAPETUM 1) operates at the apex, heterodimerizing with bHLH089 to bind TCATGTGC promoter motifs and activate downstream targets including MYB35/TDF1 and other genes that establish tapetal identity and initiate pollen wall development [104,105]. The closely related bHLH010, bHLH089, and bHLH091 act redundantly but indispensably: beyond driving target expression, they physically interact with DYT1 to increase its nuclear abundance, forming a positive feedback loop that reinforces tapetal transcription during early anther development [104]. Together with AMS (ABORTED MICROSPORES) and MYB35/TDF1, these bHLH factors form the core module ensuring the temporal precision of pollen development.

Within this cascade, bHLH010 and bHLH089 directly regulate pollen wall morphogenesis by coordinating downstream transcriptional and metabolic networks. Transcriptomic and metabolomic profiling of *bHLH010 bHLH089* double mutants revealed broad perturbations in metabolites and a reduced expression of enzymes and transporters required for pollen wall assembly [106]. A representative direct target is CSLB03, a cellulose synthase-like protein whose E-box-containing promoter is occupied by bHLH010; loss of CSLB03 phenocopies the mutant pollen wall defects, identifying it as a key downstream effector [106].

This cascade is further calibrated post-translationally. A protein phosphatase 2A (PP2A) module centered on SBI1–PP2A–C1/C5 defines the active window of bHLH089: PP2A-C1 and PP2A-C5 directly interact with bHLH089 and dephosphorylate it at Ser28 and Ser58, reducing its nuclear retention and transcriptional activity [25]. Both reduced and increased PP2A-C1/C5 activity disrupt tapetal homeostasis and impair male fertility, indicating that a precise balance between transcriptional activation and post-translational attenuation is required [25].

The DYT1–bHLH089 module also interfaces with environmental signaling. bHLH010, bHLH089, and bHLH091 safeguard pollen fertility under elevated temperature by maintaining heat-responsive gene expression during early floral development [26]. Collectively, these studies show that bHLH regulation of reproductive development operates through interconnected transcriptional cascades and post-translational mechanisms, as elaborated in the following section. In tomato (*Solanum lycopersicum*), the SlbHLH36–SlAAP8 module was recently shown to alleviate heat-induced pollen abortion by activating amino-acid transport and antioxidant systems, providing direct evidence that bHLH factors safeguard male fertility under heat in a crop species beyond *Arabidopsis* [107].

## 6. Multi-Level Regulatory Networks and Functional Integration of bHLH Transcription Factors

Plant stress responses are complex systems involving the coordinated action of multiple genes and pathways. bHLH transcription factors do not function in isolation, but together with upstream signaling pathways, downstream target genes, and other transcription factor families, constitute a precise regulatory network through four levels, namely transcriptional cascades, post-translational modifications, synergistic interactions among multiple families, and the growth–defense trade-off, thereby achieving precise and integrated regulation of plant growth, development, and stress responses.

At the transcriptional level, a typical hierarchical cascade regulatory mode exists within the bHLH family, in which upstream bHLH proteins can directly bind the promoters of downstream bHLH genes to form transcriptional cascades, achieving stepwise amplification of stress signals. The iron-deficiency response in *Arabidopsis* exemplifies this mode, in which clade IVc regulators directly activate clade Ib genes that interact with FIT to control downstream iron-uptake genes, forming a hierarchical cascade that achieves stepwise amplification of iron signals (detailed in Section 3) [21,22,24]. During stress responses, the transcriptional expression of bHLH genes is rapidly regulated by upstream signals, and different stress signals can converge on bHLH genes through different upstream pathways, achieving the integration of multiple environmental signals. Such dynamic, stress-stage- and tissue-specific expression enables bHLH genes to function as hubs that integrate converging environmental signals [101].

At the post-transcriptional and post-translational levels, bHLH activity is regulated by phosphorylation, ubiquitin-mediated degradation, changes in subcellular localization, and protein–protein interactions, enabling rapid stress responses [92]. In *Chenopodium glaucum*, CgCDPK (calcium-dependent protein kinase) directly interacts with CgbHLH001 and modulates its transcriptional activity via phosphorylation, linking calcium signaling to bHLH-mediated stress tolerance [67]. Alternative splicing and dynamic subcellular localization further contribute to this multilayered regulatory network [108].

At the level of inter-family interactions, bHLH proteins frequently converge with multiple transcription factor families, (MYB, ERF, bZIP, WRKY, and NAC) on shared stress-responsive regulons and regulatory functions, jointly contributing to a complete stress response regulatory system; however, evidence of direct protein–protein interactions between bHLH proteins and most of these families remains to be demonstrated. A representative example is the MYB-bHLH-WD40 complex, which is widely involved in multiple biological processes, including anthocyanin biosynthesis, epidermal cell development, and stress responses, and different MYB-bHLH combinations can produce different regulatory specificities [10,11]. In addition, transcription factor families such as ERF, bZIP, GRAS, WRKY, and NAC are widely involved in abiotic and biotic stress responses, and members of these families frequently converge on the same stress-responsive regulons as bHLH proteins [109,110,111,112,113]. For instance, the maize GRAS transcription factor ZmGRAS72 has been demonstrated to positively regulate drought and salt stress tolerance [114], further supporting the notion that bHLH factors cooperate with diverse TF families to form a comprehensive stress regulatory system. Direct physical and functional partnerships with bHLH proteins have been experimentally established in specific cases, most prominently within MYB-bHLH-WD40 complexes [10,11]; however, for most of these families, evidence of direct bHLH interaction remains to be demonstrated. Transcription factors play a central hub role in bridging plant biotic and abiotic stress responses, integrating different stress signals to achieve cross-regulation, and the bHLH family is an important regulatory class within this process [115].

At the level of physiological integration, bHLH transcription factors act as core hubs in the growth–defense trade-off. Under favorable conditions, HBI1 promotes growth and suppresses PAMP-triggered immunity, whereas pathogen infection inhibits HBI1 activity and activates defense at the cost of growth [9]. Recent evidence suggests that bHLH factors can also break this trade-off: in *Artemisia annua*, they may simultaneously promote biomass and the accumulation of defense-related secondary metabolites, offering new opportunities for breeding high-yield, stress-resistant crops [27].

At the level of protein–protein interactions and post-translational regulation, representative experimentally validated examples illustrate how bHLH activity is coupled to signaling and output specificity: JAZ proteins bind and inhibit MYC-family bHLH proteins in JA signaling [8,12]; MYB-bHLH-WD40 complexes activate anthocyanin biosynthetic structural genes [10,11,13]; bHLH010 directly occupies the promoter of the cellulose synthase-like gene *CSLB03* to control pollen wall formation [106]; and the SBI1–PP2A–C1/C5 module dephosphorylates bHLH089 to gate its transcriptional window during anther development [25]. In *Chenopodium glaucum*, CgCDPK phosphorylates CgbHLH001, linking calcium signaling to stress tolerance [67]. These examples show that bHLH proteins integrate diverse signaling inputs through a limited set of recurring interaction modes, and that the identity of downstream target genes determines the final physiological output.

## 7. Conclusions

After more than two decades of research, substantial progress has been made in the study of plant bHLH transcription factor families. At the fundamental level, genome-wide identification and evolutionary analyses of the bHLH family have been completed in dozens of plant species, suggesting an evolutionary pattern of “conserved domain plus variable regulatory region”, although direct experimental evidence linking regulatory-region variation to habitat-specific adaptation remains limited At the functional level, this family has been confirmed to widely participate in abiotic stresses such as low temperature, drought, salinity, and iron homeostasis, as well as in multiple biological processes including biotic stress, secondary metabolism, growth and development, and reproductive development under heat stress. At the mechanistic level, the core roles of bHLH transcription factors in hormone signaling pathways and their multi-level regulatory modes, including transcriptional cascades, post-translational modifications, and multi-factor coordination, have been progressively revealed. Related research in horticultural crops, woody plants, and cereal crops has also continued to advance, providing abundant genetic resources and theoretical support for the genetic improvement of crop stress resistance [15,16,17].

Nevertheless, current research still has clear limitations. Functional validation is highly concentrated in model plants such as *Arabidopsis* and rice, whereas studies in specialty economic crops, including tropical crops, medicinal plants, and forest trees, are dominated by expression profiling. Although a growing number of members in these species (e.g., AabHLH112, PatMYC1, DcTT8) have been functionally validated, the majority of identified family members still await in-depth mechanistic characterization. Most existing studies focus on bHLH functions under a single stress, and the regulatory mechanisms underlying combined stresses in natural environments remain unclear. Research on post-translational modifications and the dynamic regulation of subcellular localization of bHLH proteins is relatively weak, and the functional redundancy and synergistic mechanisms among different family members still require further dissection. In addition, the developmental and stress-responsive roles of bHLH proteins in reproductive tissues remain underexplored, especially under combined heat and drought conditions. Meanwhile, functional studies of most genes remain at the laboratory stage, with insufficient translation into crop breeding and a lack of superior alleles that can be directly used in breeding practice [18,19,20].

Critical evaluation of the literature also reveals contradictions that remain unresolved. For example, SbbHLH85 promotes root-hair elongation yet reduces salt tolerance through Na^+^ overaccumulation, illustrating that a single bHLH member can exert context-dependent positive and negative effects [50]. PIF-family members integrate light signals with growth promotion, yet their roles in stress defense are highly context-dependent [70,71], and the growth–defense trade-off exemplified by HBI1 can be overcome in specific systems, such as *Artemisia annua* [27]. The frequent reliance on genome-wide expression profiling rather than genetic and biochemical validation in non-model species further limits the strength of the causal conclusions that can currently be drawn. These contradictory and unresolved aspects are important targets for future research.

Future research can be advanced from four directions. First, multi-omics technologies and high-throughput functional identification methods should be used to systematically dissect the global regulatory network of bHLH transcription factors, clarify the upstream–downstream relationships and functional redundancy among different family members, and construct a complete regulatory atlas; single-cell transcriptomics should be applied to resolve the cell-type-specific regulatory logic of bHLH factors in stress-responsive tissues and reproductive organs. Second, functional studies under combined stresses should be strengthened to dissect the integrative roles of bHLH transcription factors in cross-responses to multiple stresses, more closely reflecting actual field production scenarios. Third, research on post-translational modifications and dynamic protein regulation should be deepened to reveal the rapid regulatory mechanisms of bHLH activity at the protein level and to refine the multi-level regulatory network. Fourth, the functional mining and breeding application of bHLH transcription factors in specialty economic crops should be enhanced. Gene editing technologies (e.g., CRISPR/Cas) offer a promising but still experimental route to edit target genes for the improvement of crop stress resistance and quality traits; their successful deployment will require the systematic evaluation of pleiotropy, functional redundancy, and field performance, as well as the mining of superior alleles, before translation into production applications. In parallel, AI-assisted prediction of bHLH regulatory networks, integrating transcriptomic, epigenomic, and protein-interaction datasets, is expected to accelerate the identification of high-priority targets for functional validation and breeding.

## Figures and Tables

**Table 1:** Genome-wide identification of the bHLH transcription factor family across plant species.

Type	Species	Main Research Focus	Key Functional Genes	Validation Status	References
Model plants	*Arabidopsis thaliana*	Family classification, abiotic stress, hormone signaling	*bHLH34*, *bHLH104*, *bHLH115*, *HBI1*, *AtbHLH68*	Functionally validated: AtbHLH68, HBI1, bHLH34, bHLH104, bHLH115	[5,6,7,20,21,22,46]
	*Oryza sativa*	Cold stress, iron homeostasis, light signaling	*OsbHLH1*, *IRO2*, *OsbHLH057*, *OsbHLH064*	Functionally validated: IRO2, OsbHLH057, OsbHLH064; expression-based: OsbHLH1	[1,2,47,48]
Cereal crops	*Triticum aestivum*	Genome-wide identification, abiotic stress expression	*TabHLH12*, *TabHLH56*	Expression-based candidates	[30,31,32]
	*Zea mays*	Evolutionary analysis, drought response	*ZmPTF1*	Functionally validated (ZmPTF1)	[4,49]
	*Sorghum bicolor*	Salt stress, root hair development	*SbbHLH85*	Functionally validated (SbbHLH85)	[50]
	*Avena sativa*	Abiotic stress expression profiling	*AsbHLH23*, *AsbHLH78*	Expression-based candidates	[35]
	*Secale cereale*	Phylogeny and expression analysis	*ScbHLH17*, *ScbHLH49*	Expression-based candidates	[37]
	*Brachypodium distachyon*	Genome-wide identification, salt stress	*-*	Expression-based candidates	[36]
Legumes	*Glycine max*	Abiotic stress, jasmonate regulation	*GmbHLH3*	Functionally validated (GmbHLH3)	[3,33]
	*Medicago truncatula*	Triterpene saponin biosynthesis	*TSAR1*, *TSAR2*	Functionally validated (TSAR1, TSAR2)	[51]
	*Medicago sativa*	Cold stress expression profiling	*MsbHLH18*, *MsbHLH67*	Expression-based candidates	[52]
Horticultural crops	*Solanum lycopersicum*	Drought/salt stress, iron homeostasis	*SlbHLH96*, *SlbHLH152*	Functionally validated (SlbHLH96, SlbHLH152)	[53,54]
	*Cucumis sativus*	Heat stress resistance	*CsSPT*	Functionally validated (CsSPT)	[55]
	*Citrus sinensis*	Citrus canker resistance	*CsbHLH085*	Functionally validated (CsbHLH085)	[56]
Fruit crops	*Malus domestica*	Drought/salt stress, water resistance	*MdbHLH130*, *MdCIB1*	Functionally validated (MdbHLH130, MdCIB1)	[57,58]
	*Prunus avium*	Cold stress expression profiling	*PavbHLH16*, *PavbHLH42*	Expression-based candidates	[59]
	*Mangifera indica*	Family characterization, expression patterns	*MibHLH21*, *MibHLH58*	Expression-based candidates	[41]
	*Dimocarpus longan*	Flowering regulation	*DlbHLH8*	Functionally validated (DlbHLH8)	[40]
Woody plants	*Populus trichocarpa*	Drought, salt stress	*PtbHLH*, *PxbHLH02*	Functionally validated: PxbHLH02; expression-based: PtbHLHs	[60,61]
	*Broussonetia papyrifera*	Cold stress transcriptional response	*BpbHLH09*, *BpbHLH35*	Expression-based candidates	[62]
	*Prunus sibirica*	Cold stress response, functional prediction	*PsbHLH14*, *PsbHLH52*	Expression-based candidates	[63]
Medicinal plants	*Panax ginseng*	Secondary metabolism prediction	*PgbHLH25*, *PgbHLH61*	Prediction/expression-based candidates	[42]
	*Dendrobium candidum*	Anthocyanin biosynthesis	*DcTT8*	Functionally validated (DcTT8)	[13]
	*Artemisia annua*	Sesquiterpene synthesis, growth–defense balance	*AabHLH112*	Functionally validated (AabHLH112)	[27,64]
	*Pogostemon cablin*	Sesquiterpene synthesis, jasmonate response	*PatMYC1*	Functionally validated (PatMYC1)	[65]
Stress-tolerant plants	*Chenopodium glaucum*	Drought response, protein interaction	*CgbHLH001*	Functionally validated (CgbHLH001)	[66,67]
	*Agriophyllum squarrosum*	Development and abiotic stress response	*AsqbHLH08*, *AsqbHLH33*	Expression-based candidates	[45]
	*Pseudoroegneria libanotica*	Drought stress during germination	*PlbHLH11*, *PlbHLH37*	Expression-based candidates	[44]

**Table 3:** Key bHLH transcription factors involved in secondary metabolism and biotic stress responses.

Type	Gene	Species	Encoded Protein	Expression Pattern	Regulatory Mechanism	Ref.
Anthocyanin metabolism	*Gentian bHLH*	*Gentiana*	bHLH TF	High expression in petals during flower development	Synergizes with R2R3-MYB to regulate anthocyanin biosynthesis	[94]
	*DcTT8*	*Dendrobium candidum*	bHLH TF	Expressed in pigment-accumulating tissues (flowers, stems)	Directly binds promoters of key anthocyanin biosynthetic genes	[13]
	*CsbHLH89*	*Camellia sinensis*	Light-responsive bHLH	Upregulated in leaves under light induction	Mediates light signaling, positively regulates anthocyanin accumulation in tea	[73]
Terpenoid metabolism	*TSAR1/TSAR2*	*Medicago truncatula*	bHLH TF	High expression in triterpene saponin-producing tissues	Specifically activates key genes in the triterpene saponin biosynthetic pathway	[51]
	*AabHLH112*	*Artemisia annua*	bHLH TF	High expression in *Artemisia annua* glandular trichomes	Positively regulates sesquiterpene synthesis (epi-cedrol, beta-farnesene)	[64]
Biotic stress	*HBI1*	*Arabidopsis thaliana*	bHLH TF	High expression in actively growing tissues	Mediates trade-off between growth and PAMP-triggered immunity	[9]
	*CsbHLH085*	*Citrus sinensis*	bHLH TF	Upregulated in peel under pathogen infection	Activates defense genes and phytoalexin synthesis, enhances canker resistance	[56]

## Data Availability

No new data were created or analyzed in this study. Data sharing is not applicable to this article.

## References

[1] Wang Y.J., Zhang Z.G., He X.J., Zhou H.-L., Wen Y.-X., Dai J.-X., Zhang J.-S., Chen S.-Y. (2003). A rice transcription factor OsbHLH1 is involved in cold stress response. Theor. Appl. Genet..

[2] Ogo Y., Itai R.N., Nakanishi H., Inoue H., Kobayashi T., Suzuki M., Takahashi M., Mori S., Nishizawa N.K. (2006). Isolation and characterization of IRO2, a novel iron-regulated bHLH transcription factor in *graminaceous* plants. J. Exp. Bot..

[3] Osorio M., Bücker-Neto L., Castilhos G., Turchetto-Zolet A.C., Wiebke-Strohm B., Bodanese-Zanettini M.H., Margis-Pinheiro M. (2012). Identification and in silico characterization of soybean trihelix-GT and bHLH transcription factors involved in stress responses. Genet. Mol. Biol..

[4] Zhang T.T., Lv W., Zhang H.S., Ma L., Li P., Ge L., Li G. (2018). Genome-wide analysis of the basic Helix-Loop-Helix (bHLH) transcription factor family in maize. BMC Plant Biol..

[5] Heim M.A., Jakoby M., Werber M., Martin C., Weisshaar B., Bailey P.C. (2003). The Basic Helix-Loop-Helix Transcription Factor Family in Plants: A Genome-Wide Study of Protein Structure and Functional Diversity. Mol. Biol. Evol..

[6] Gao F., Dubos C. (2024). The *Arabidopsis* bHLH transcription factor family. Trends Plant Sci..

[7] Carretero-Paulet L., Galstyan A., Roig-Villanova I., Martínez-García J.F., Bilbao-Castro J.R., Robertson D.L. (2010). Genome-Wide Classification and Evolutionary Analysis of the bHLH Family of Transcription Factors in *Arabidopsis*, Poplar, Rice, Moss, and Algae. Plant Physiol..

[8] Niu Y.J., Figueroa P., Browse J. (2011). Characterization of JAZ-interacting bHLH transcription factors that regulate jasmonate responses in *Arabidopsis*. J. Exp. Bot..

[9] Fan M., Bai M.Y., Kim J.G., Wang T., Oh E., Chen L., Park C.H., Son S.-H., Kim S.-K., Mudgett M.B. (2014). The bHLH Transcription Factor HBI1 Mediates the Trade-Off between Growth and Pathogen-Associated Molecular Pattern–Triggered Immunity in *Arabidopsis*. Plant Cell.

[10] Feller A., Machemer K., Braun E., Grotewold E. (2010). Evolutionary and comparative analysis of MYB and bHLH plant transcription factors. Plant J..

[11] Wang B.H., Luo Q., Li Y.P., Du K., Wu Z., Li T., Shen W.-H., Huang C.-H., Gan J., Dong A. (2022). Structural insights into partner selection for MYB and bHLH transcription factor complexes. Nat. Plants.

[12] Goossens J., Mertens J., Goossens A. (2016). Role and functioning of bHLH transcription factors in jasmonate signalling. J. Exp. Bot..

[13] Jia N., Wang J.J., Liu J.M., Jiang J., Sun J., Yan P., Sun Y., Wan P., Ye W., Fan B. (2021). DcTT8, a bHLH transcription factor, regulates anthocyanin biosynthesis in *Dendrobium candidum*. Plant Physiol. Biochem..

[14] Lei P., Jiang Y.X., Zhao Y., Jiang M., Ji X., Ma L., Jin G., Li J., Zhang S., Kong D. (2024). Functions of Basic Helix–Loop–Helix (bHLH) Proteins in the Regulation of Plant Responses to Cold, Drought, Salt, and Iron Deficiency: A Comprehensive Review. J. Agric. Food Chem..

[15] Wang K.N., Liu H.Y., Mei Q.L., Yang J., Ma F., Mao K. (2022). Characteristics of bHLH transcription factors and their roles in the abiotic stress responses of horticultural crops. Sci. Hortic..

[16] Song S., Zhang N.N., Fan X.W., Wang G. (2025). bHLH Transcription Factors in Cereal Crops: Diverse Functions in Regulating Growth, Development and Stress Responses. Int. J. Mol. Sci..

[17] Yan T.Y., Shu X.C., Ning C.L., Li Y., Wang Z., Wang T., Zhuang W. (2024). Functions and Regulatory Mechanisms of bHLH Transcription Factors during the Responses to Biotic and Abiotic Stresses in Woody Plants. Plants.

[18] Guo J.R., Sun B.X., He H.R., Zhang Y., Tian H., Wang B. (2021). Current Understanding of bHLH Transcription Factors in Plant Abiotic Stress Tolerance. Int. J. Mol. Sci..

[19] Zuo Z.F., Lee H.Y., Kang H.G. (2023). Basic Helix-Loop-Helix Transcription Factors: Regulators for Plant Growth Development and Abiotic Stress Responses. Int. J. Mol. Sci..

[20] Qian Y.C., Zhang T.Y., Yu Y., Gou L.P., Yang J.T., Xu J., Pi E.X. (2021). Regulatory Mechanisms of bHLH Transcription Factors in Plant Adaptive Responses to Various Abiotic Stresses. Front. Plant Sci..

[21] Liang G., Zhang H.M., Li X.L., Ai Q., Yu D. (2017). bHLH transcription factor bHLH115 regulates iron homeostasis in *Arabidopsis thaliana*. J. Exp. Bot..

[22] Li X.L., Zhang H.M., Ai Q., Liang G., Yu D. (2016). Two bHLH Transcription Factors, bHLH34 and bHLH104, Regulate Iron Homeostasis in *Arabidopsis thaliana*. Plant Physiol..

[23] Zhang Z.X., Cheng J., Wang W.X., Gao Y., Xian X., Li C., Wang Y. (2023). Transcription factors dealing with Iron-deficiency stress in plants: Focus on the bHLH transcription factor family. Physiol. Plant..

[24] Gao F., Robe K., Gaymard F., Izquierdo E., Dubos C. (2019). The Transcriptional Control of Iron Homeostasis in Plants: A Tale of bHLH Transcription Factors?. Front. Plant Sci..

[25] Lai Z., Lei Z., Wang J., Liu Y., Fu Y., Chang F. (2026). A PP2A phosphatase-defined transcriptional activity window safeguards male fertility. Plant Cell.

[26] Fu Y., Yang T., Lin S., Bao W., Liu P., Yu X., Wang C., Chen L., Diao Z., Cui J. (2020). Analyses of functional conservation and divergence reveal requirement of bHLH010/089/091 for pollen development at elevated temperature in *Arabidopsis*. J. Genet. Genom..

[27] Yuan M.Y., Zhou F. (2026). Breaking the Growth-Defense Trade-Off: bHLH Transcription Factors as Integrators of Development, Metabolism, and Yield in *Artemisia annua*. Genes.

[28] Zhang Z.L. (2011). The Structure and Function of Plant bHLH Transcription Factors. J. Trop. Subtrop. Bot..

[29] Castelain M., Le Hir R., Bellini C. (2012). The non-DNA-binding bHLH transcription factor PRE3/bHLH135/ATBS1/TMO7 is involved in the regulation of light signaling pathway in *Arabidopsis*. Physiol. Plant..

[30] Wei K., Chen H. (2018). Comparative functional genomics analysis of bHLH gene family in rice, maize and wheat. BMC Plant Biol..

[31] Guo X.J., Wang J.R. (2017). Global identification, structural analysis and expression characterization of bHLH transcription factors in wheat. BMC Plant Biol..

[32] Wang L.Z., Xiang L.J., Hong J., Xie Z., Li B. (2019). Genome-wide analysis of bHLH transcription factor family reveals their involvement in biotic and abiotic stress responses in wheat (*Triticum aestivum* L.). 3 Biotech.

[33] Wang T.L., Wang S., Wang Y., Li J., Yan F., Liu Y., Zhao L., Wang Q. (2020). Jasmonic acid–induced inhibition of root growth and leaf senescence is reduced by GmbHLH3, a soybean bHLH transcription factor. Can. J. Plant Sci..

[34] Wu N., Jiang T., Feng Y., Yuan M. (2025). Genome-Wide Identification and Expression Analysis of Soybean bHLH Transcription Factor and Its Molecular Mechanism on Grain Protein Synthesis. Front. Plant Sci..

[35] Leng H.J., Zhang X.P., Zhang T., Cui J., Zhang Y., Dai H., Zhang L., Liu H., Wang X., Kang J. (2026). Genome-wide identification and abiotic stress response analysis of the oat bHLH gene family. Front. Plant Sci..

[36] Niu X., Guan Y.X., Chen S.K., Li H. (2017). Genome-wide analysis of basic helix-loop-helix (bHLH) transcription factors in *Brachypodium distachyon*. BMC Genom..

[37] Chen X.Y., Yao C.M., Liu J.H., Fang J., Deng H., Yao Q., Kang T., Guo X. (2024). Basic helix-loop-helix (bHLH) gene family in rye (*Secale cereale* L.): Genome-wide identification, phylogeny, evolutionary expansion and expression analyses. BMC Genom..

[38] Mao K., Dong Q.L., Li C., Ma F. (2017). Genome Wide Identification and Characterization of Apple bHLH Transcription Factors and Expression Analysis in Response to Drought and Salt Stress. Front. Plant Sci..

[39] Zhang J.L., Liu X.Y., Yin Z.Z., Zhao T., Du D., Li J., Zhu M., Sun Y., Pan Y. (2025). Genome- and Transcriptome-Wide Characterization and Expression Analyses of bHLH Transcription Factor Family Reveal Their Relevance to Salt Stress Response in Tomato. Plants.

[40] Liu Y., Sang X.L., Liu L.Q., Ren C., Zhang Z., Chen T., Li H., Jue D., Shi S. (2026). Whole genome identification and operational analysis of the bHLH gene family in longan and the role of DlbHLH8 in flowering regulation. Front. Plant Sci..

[41] Salih H., Tan L., Htet N. (2021). Genome-Wide Identification, Characterization of bHLH Transcription Factors in Mango. Trop. Plant Biol..

[42] Chu Y., Xiao S.M., Su H., Liao B., Zhang J., Xu J., Chen S. (2018). Genome-wide characterization and analysis of bHLH transcription factors in *Panax ginseng*. Acta Pharm. Sin. B.

[43] Sun Y.X., Wang B.E., Zhang L.C., Zheng X., Xu P., Zhang M., Han M., Di P., Han M., Cheng L. (2025). Genome-wide identification of the bHLH gene family in *Scutellaria baicalensis* and their relationship with baicalin biosynthesis under drought stress. Front. Plant Sci..

[44] Zhai X.G., Wang X., Yang X.Z., Huang Q., Wu D., Wang Y., Kang H., Sha L., Fan X., Zhou Y. (2024). Genome-wide identification of bHLH transcription factors and expression analysis under drought stress in *Pseudoroegneria libanotica* at germination. Physiol. Mol. Biol. Plants.

[45] Zhang R.Z., Cui X.Y., Li X.F., Zhao P. (2025). Genome-wide identification of bHLH transcription factors related to plant development and abiotic stress response in sand rice (*Agriophyllum squarrosum* (L.) Moq.). Genetica.

[46] Hussain Q., Asim M., Zhang R., Khan R., Farooq S., Wu J. (2021). Transcription Factors Interact with ABA through Gene Expression and Signaling Pathways to Mitigate Drought and Salinity Stress. Biomolecules.

[47] Wang W.J., Shinwari K., Zhang H., Zhang H., Dong L., He F., Zheng L. (2022). The bHLH Transcription Factor OsbHLH057 Regulates Iron Homeostasis in Rice. Int. J. Mol. Sci..

[48] Gao F., Zhu Z.K., Xue K.X., Fu Z., Yue J., Zhang N., Zhou H., Deng Y., Zhou J., Kuang L. (2026). OsbHLH064, an IVb bHLH Transcription Factor, Regulates Iron Homeostasis and Enhances Grain Fe Accumulation in Rice. Plant Biotechnol. J..

[49] Li Z.X., Liu C., Zhang Y., Wang B., Ran Q., Zhang J. (2019). The bHLH family member ZmPTF1 regulates drought tolerance in maize by promoting root development and abscisic acid synthesis. J. Exp. Bot..

[50] Song Y.S., Li S.M., Sui Y., Zheng H., Han G., Sun X., Yang W., Wang H., Zhuang K., Kong F. (2021). SbbHLH85, a bHLH member, modulates resilience to salt stress by regulating root hair growth in sorghum. Theor. Appl. Genet..

[51] Mertens J., Pollier J., Vanden Bossche R., Lopez-Vidriero I., Franco-Zorrilla J.M., Goossens A. (2015). The bHLH Transcription Factors TSAR1 and TSAR2 Regulate Triterpene Saponin Biosynthesis in *Medicago truncatula*. Plant Physiol..

[52] Li G.J., Jin L., Sheng S. (2022). Genome-Wide Identification of bHLH Transcription Factor in *Medicago sativa* in Response to Cold Stress. Genes.

[53] Liang Y.F., Ma F., Li B.Y., Guo C., Hu T., Zhang M., Liang Y., Zhu J., Zhan X. (2022). A bHLH transcription factor, SlbHLH96, promotes drought tolerance in tomato. Hortic. Res..

[54] Li X.L., Cao H.H., Yu D.S., Xu K., Zhang Y., Shangguan X., Zheng X., Yang Z., Li C., Pan X. (2023). SlbHLH152, a bHLH transcription factor positively regulates iron homeostasis in tomato. Plant Sci..

[55] Liang Y.G., Yang C.Y., Ming F.Y., Yu B., Cheng Z., Wang Y., Qiu Z., Zhang X., Cao B., Yan S. (2024). A bHLH transcription factor, CsSPT, regulates high-temperature resistance in cucumber. Hortic. Plant J..

[56] Huang X., Su L.Y., Xian B.H., Yu Q., Zhang M., Fan J., Zhang C., Liu Y., He H., Zhong X. (2024). Genome-wide identification and characterization of the sweet orange (*Citrus sinensis*) basic helix-loop-helix (bHLH) family reveals a role for CsbHLH085 as a regulator of citrus bacterial canker resistance. Int. J. Biol. Macromol..

[57] Zhao Q., Fan Z.H., Qiu L.N., Che Q., Wang T., Li Y., Wang Y. (2020). MdbHLH130, an Apple bHLH Transcription Factor, Confers Water Stress Resistance by Regulating Stomatal Closure and ROS Homeostasis in Transgenic Tobacco. Front. Plant Sci..

[58] Ren Y.R., Yang Y.Y., Zhao Q., Zhang T.-E., Wang C.-K., Hao Y.-J., You C.-X. (2021). MdCIB1, an apple bHLH transcription factor, plays a positive regulator in response to drought stress. Environ. Exp. Bot..

[59] Shen T.J., Wen X.P., Wen Z., Qiu Z., Hou Q., Li Z., Mei L., Yu H., Qiao G. (2021). Genome-wide identification and expression analysis of bHLH transcription factor family in response to cold stress in sweet cherry (*Prunus avium* L.). Sci. Hortic..

[60] Gao S.Z., Li C.H., Chen X.H., Li S., Liang N., Wang H., Zhan Y., Zeng F. (2023). Basic helix-loop-helix transcription factor PxbHLH02 enhances drought tolerance in *Populus* (*Populus simonii* × *P. nigra*). Tree Physiol..

[61] Zhang H.Z., Ye S.H., Wang N., Xu Z., Gong S. (2024). Analyses of the bHLH gene family in *Populus trichocarpa* reveal roles of four PtbHLHs in regulating the drought stress response. Environ. Exp. Bot..

[62] Peng X.J., Wu Q.Q., Teng L.H., Tang F., Pi Z., Shen S. (2015). Transcriptional regulation of the paper mulberry under cold stress as revealed by a comprehensive analysis of transcription factors. BMC Plant Biol..

[63] Liu Q.G., Wen J.X., Wang S.P., Chen J., Sun Y., Liu Q., Li X., Dong S. (2023). Genome-wide identification, expression analysis, and potential roles under low-temperature stress of bHLH gene family in *Prunus sibirica*. Front. Plant Sci..

[64] Xiang L.N., He P., Shu G.P., Yuan M., Wen M., Lan X., Liao Z., Tang Y. (2022). AabHLH112, a bHLH transcription factor, positively regulates sesquiterpenes biosynthesis in *Artemisia annua*. Front. Plant Sci..

[65] Zhang P.X., Wang Y.S., Li M.K., Li J., Jia Y., Yan H., Xia J., Xia K., He M. (2026). PatMYC1, a JA-responsive bHLH transcription factor, regulates sesquiterpene biosynthesis by activating the MVA pathway in *Pogostemon cablin*. Ind. Crops Prod..

[66] Zhao H.J., Wang C.H., Lan H.Y. (2021). A bHLH transcription factor from *Chenopodium glaucum* confers drought tolerance to transgenic maize by positive regulation of morphological and physiological performances and stress-responsive genes’ expressions. Mol. Breed..

[67] Zhou Z.X., Wang J., Zhang S.Y., Yu Q., Lan H. (2021). Investigation of the Nature of CgCDPK and CgbHLH001 Interaction and the Function of bHLH Transcription Factor in Stress Tolerance in *Chenopodium glaucum*. Front. Plant Sci..

[68] Chang G.X., Ma J.C., Wang S.H., Tang M., Zhang B., Ma Y., Li L., Sun G., Dong S., Liu Y. (2023). Liverwort bHLH transcription factors and the origin of stomata in plants. Curr. Biol..

[69] Ballester P., Martínez-Godoy M., Ezquerro M., Navarrete-Gómez M., Trigueros M., Rodríguez-Concepción M., Ferrándiz C. (2021). A transcriptional complex of NGATHA and bHLH transcription factors directs stigma development in *Arabidopsis*. Plant Cell.

[70] Duek P., Fankhauser C. (2005). bHLH class transcription factors take centre stage in phytochrome signalling. Trends Plant Sci..

[71] Sun Y.X., Li Q., Wu M.D., Wang Q., Zhang D., Gao Y. (2024). Rice PIFs: Critical regulators in rice development and stress response. Plant Mol. Biol..

[72] Jiang S.H., Jin H.Y., Tian N., Liu S. (2025). Expression pattern and transcriptional regulation of CsPIF7 in *Camellia sinensis*. Chin. J. Biotechnol..

[73] Zhang K.K., Lin C.Y., Chen B.Y., Lin Y., Su H., Du Y., Zhang H., Zhou H., Ji R., Zhang L. (2023). A light responsive transcription factor CsbHLH89 positively regulates anthocyanidin synthesis in tea (*Camellia sinensis*). Sci. Hortic..

[74] Chinnusamy V., Ohta M., Kanrar S., Lee B.-H., Hong X., Agarwal M., Zhu J.-K. (2003). ICE1: A regulator of cold-induced transcriptome and freezing tolerance in *Arabidopsis*. Genes Dev..

[75] Li R.X., Ahmad B., Hwarari D., Li D., Lu Y., Gao M., Chen J., Yang L. (2022). Genomic Survey and Cold-Induced Expression Patterns of bHLH Transcription Factors in *Liriodendron chinense* (*Hemsl*.) Sarg. Forests.

[76] Pan J., Yang Z., Liu S., Liu Z., Yu T. (2026). The bHLH Transcription Factor IbbHLH129 Positively Regulates the Cold Tolerance of *Sweetpotato* Seedlings by Modulating Auxin and Gibberellin Pathways. Plants.

[77] Kim J., Kim H.Y. (2006). Molecular characterization of a bHLH transcription factor involved in *Arabidopsis* abscisic acid-mediated response. Biochim. Biophys. Acta Gene Struct. Expr..

[78] Le Hir R., Castelain M., Chakraborti D., Moritz T., Dinant S., Bellini C. (2017). AtbHLH68 transcription factor contributes to the regulation of ABA homeostasis and drought stress tolerance in *Arabidopsis thaliana*. Physiol. Plant..

[79] Gu X.Y., Gao S.X., Li J., Song P., Zhang Q., Guo J., Wang X., Han X., Wang X., Zhu Y. (2021). The bHLH transcription factor regulated gene OsWIH2 is a positive regulator of drought tolerance in rice. Plant Physiol. Biochem..

[80] Li C.J., Yan C.X., Sun Q.X., Wang J., Yuan C., Mou Y., Shan S., Zhao X. (2021). The bHLH transcription factor AhbHLH112 improves the drought tolerance of peanut. BMC Plant Biol..

[81] Kiribuchi K., Jikumaru Y., Kaku H., Minami E., Hasegawa M., Kodama O., Seto H., Okada K., Nojiri H., Yamane H. (2005). Involvement of the Basic Helix-Loop-Helix Transcription Factor RERJ1 in Wounding and Drought Stress Responses in Rice Plants. Biosci. Biotechnol. Biochem..

[82] Zhao X.B., Wang Q., Yan C.X., Sun Q., Wang J., Li C., Yuan C., Mou Y., Shan S. (2023). The bHLH transcription factor *AhbHLH121* improves salt tolerance in peanut. Int. J. Biol. Macromol..

[83] Li L., Zhao Z.Z., Li W.Q., Wang Q., Wang Y., Ji X. (2025). The bHLH Transcription Factor PdbUNE12 Functions as a Positive Regulator of the Salt Stress Response in *Populus davidiana* × *Populus bolleana*. Physiol. Plant..

[84] Zhang Z.J., Fang J., Zhang L., Jin H., Fang S. (2023). Genome-wide identification of bHLH transcription factors and their response to salt stress in *Cyclocarya paliurus*. Front. Plant Sci..

[85] Lei P., Liu Z., Hu Y.B., Kim H., Liu S., Liu J., Xu L., Li J., Zhao Y., Yu Z. (2021). Transcriptome analysis of salt stress responsiveness in the seedlings of wild and cultivated *Ricinus communis* L.. J. Biotechnol..

[86] Ni L.J., Wang Z.Q., Fu Z.K., Liu D., Yin Y., Li H., Gu C. (2021). Genome-wide Analysis of Basic Helix-Loop-Helix Family Genes and Expression Analysis in Response to Drought and Salt Stresses in *Hibiscus hamabo* Sieb. et Zucc. Int. J. Mol. Sci..

[87] Ling H.Q., Bauer P., Bereczky Z., Keller B., Ganal M. (2002). The tomato fer gene encoding a bHLH protein controls iron-uptake responses in dicot plants. Proc. Natl. Acad. Sci. USA.

[88] Li C., Zhao J., Ping H., Li Y., Lei R., Pan B., Liang G. (2025). OsbHLH062 negatively regulates Fe homeostasis by enhancing OsHRZ1 targeting OsPRIs for degradation. New Phytol..

[89] Cao Z.D., Wang E.B., Zhai M.H., Li Y., Li Y., Shi J., Feng X., Wang J., Song X., Shi Y. (2025). Identification of bHLH Family Genes in *Rhododendron hainanense* Merr. and Verification of Their Role in Heat Tolerance. Forests.

[90] Wang C., Tu S.H., Zou L.P., Wang X., Li C. (2025). Comprehensive analysis of bHLH transcription factors reveals candidate regulators of flower development and heat stress response in *Rhododendron simsii*. BMC Plant Biol..

[91] Zhang H.W., Lu L.L. (2024). Transcription factors involved in plant responses to cadmium-induced oxidative stress. Front. Plant Sci..

[92] Pireyre M., Burow M. (2015). Regulation of MYB and bHLH Transcription Factors: A Glance at the Protein Level. Mol. Plant.

[93] Lu R., Zhang J., Wu Y.W., Wang Y., Zhang J., Zheng Y., Li Y., Li X.-B. (2021). bHLH transcription factors LP1 and LP2 regulate longitudinal cell elongation. Plant Physiol..

[94] Nakatsuka T., Haruta K., Pitaksutheepong C., Abe Y., Kakizaki Y., Yamamoto K., Shimada N., Yamamura S., Nishihara M. (2008). Identification and Characterization of R2R3-MYB and bHLH Transcription Factors Regulating Anthocyanin Biosynthesis in *Gentian* Flowers. Plant Cell Physiol..

[95] Liu Q., Zhao Y.Z., Wang Y., Wei Q., Zhang Y., Li S. (2025). Comprehensive genome-wide analysis of bHLH family genes in *Nymphaea colorata* and molecular characterization of NcTT8. BMC Plant Biol..

[96] Yang S., Liu M., Zhao C., Wang R., Xue L., Lei J. (2025). A novel bHLH transcription factor, FabHLH110, is involved in regulation of anthocyanin synthesis in petals of pink-flowered strawberry. Plant Physiol. Biochem..

[97] Niu Z., Zhang Z., Zhao Y., Xuan L., Chen Z., Yang L. (2025). Transcription Factor VvbHLH137 Positively Regulates Anthocyanin Accumulation in Grape (*Vitis vinifera*). Plants.

[98] Arshad K., Li C.H., Li L.S., Wang J., Chen J., Zhao Y. (2025). Genome-wide identification and expression profiling of bHLH transcription factors associated with ferulic acid biosynthesis in *Angelica sinensis*. Front. Plant Sci..

[99] Meraj T., Fu J.Y., Raza M., Zhu C., Shen Q., Xu D., Wang Q. (2020). Transcriptional Factors Regulate Plant Stress Responses Through Mediating Secondary Metabolism. Genes.

[100] Ng D., Abeysinghe J., Kamali M. (2018). Regulating the Regulators: The Control of Transcription Factors in Plant Defense Signaling. Int. J. Mol. Sci..

[101] Sun X., Wang Y., Sui N. (2018). Transcriptional regulation of bHLH during plant response to stress. Biochem. Biophys. Res. Commun..

[102] Ariizumi T., Toriyama K. (2011). Genetic regulation of sporopollenin synthesis and pollen exine development. Annu. Rev. Plant Biol..

[103] Clement C., Audran J.C. (1995). Anther wall layers control pollen sugar nutrition in Lilium. Protoplasma.

[104] Cui J., You C., Zhu E., Huang Q., Ma H., Chang F. (2016). Feedback regulation of DYT1 by interactions with downstream bHLH factors promotes DYT1 nuclear localization and anther development. Plant Cell.

[105] Feng B., Lu D., Ma X., Peng Y., Sun Y., Ning G., Ma H. (2012). Regulation of the *Arabidopsis* anther transcriptome by DYT1 for pollen development. Plant J..

[106] Lai Z., Wang J., Peng S.-Q., Chang F. (2022). bHLH010/089 Transcription Factors Control Pollen Wall Development via Specific Transcriptional and Metabolic Networks in *Arabidopsis thaliana*. Int. J. Mol. Sci..

[107] Ding X., Qiang Q., Xu G., Zhao W., Zhang Z., Wang C., Ou X., Yang J., Wang S., Yang J. (2026). SlbHLH36–SlAAP8 alleviates heat-induced pollen abortion by regulating amino acid metabolism in tomato. J. Genet. Genom..

[108] Radani Y., Li R.X., Korboe H., Ma H., Yang L. (2023). Transcriptional and Post-Translational Regulation of Plant bHLH Transcription Factors during the Response to Environmental Stresses. Plants.

[109] Wu Y., Li X., Zhang J.N., Zhao H., Tan S., Xu W., Pan J., Yang F., Pi E. (2022). ERF subfamily transcription factors and their function in plant responses to abiotic stresses. Front. Plant Sci..

[110] Xiong H.Y., He H.D., Chang Y., Miao B., Liu Z., Wang Q., Dong F., Xiong L. (2025). Multiple roles of NAC transcription factors in plant development and stress responses. J. Integr. Plant Biol..

[111] Liu H.T., Tang X., Zhang N., Li S., Si H. (2023). Role of bZIP Transcription Factors in Plant Salt Stress. Int. J. Mol. Sci..

[112] Neves C., Ribeiro B., Amaro R., Expósito J., Grimplet J., Fortes A.M. (2023). Network of GRAS transcription factors in plant development, fruit ripening and stress responses. Hortic. Res..

[113] Wang H.L., Cheng X., Yin D.M., Chen D., Luo C., Liu H., Huang C. (2023). Advances in the Research on Plant WRKY Transcription Factors Responsive to External Stresses. Curr. Issues Mol. Biol..

[114] She M., Zheng D., Zhang S., Ke Z., Wu Z., Zou H., Zhang Z. (2024). Functional Analysis of Maize GRAS Transcription Factor Gene ZmGRAS72 in Response to Drought and Salt Stresses. Agric. Commun..

[115] Liu F., Xi M.W., Liu T., Wu X., Ju L., Wang D. (2023). The central role of transcription factors in bridging biotic and abiotic stress responses for plants’ resilience. New Crops.

